# Synergistic photothermal ablative effects of functionalizing carbon nanotubes with a POSS-PCU nanocomposite polymer

**DOI:** 10.1186/1477-3155-10-34

**Published:** 2012-07-31

**Authors:** Aaron Tan, Seyed Yazdan Madani, Jayakumar Rajadas, Giorgia Pastorin, Alexander M Seifalian

**Affiliations:** 1 Centre for Nanotechnology & Regenerative Medicine, UCL Division of Surgery & Interventional Science, University College London, London, NW3 2QG, UK; 2 Department of Neurology & Neurological Sciences, Biomaterials & Advanced Drug Delivery Laboratory, School of Medicine, Stanford University, California, USA; 3 Department of Pharmacy, Faculty of Science, National University of Singapore, Singapore, Singapore; 4 Royal Free London NHS Foundation Trust Hospital, London, UK

**Keywords:** Carbon nanotubes, POSS-PCU, Thermal ablation, Nanotechnology, Nanocomposite polymer, Colorectal cancer

## Abstract

**Background:**

The application of nanotechnology in biology and medicine represents a significant paradigm shift in the approach to the treatment of cancer. Evidence suggests that when exposed to near-infrared radiation (NIR), carbon nanotubes (CNTs) dissipate a substantial amount of heat energy. We have developed a novel nanocomposite polymer, polyhedral oligomeric silsesquioxane poly (carbonate-urea) urethane (POSS-PCU). POSS-PCU displays excellent biocompatibility and has been used in making artificial organs as well as protective coatings for medical devices.

**Results:**

Functionalizing (or “coating”) CNTs with POSS-PCU confers biocompatibility and increase the amount of heat energy generated, by enhancing dispersion. Here we demonstrate that POSS-PCU-functionalized multi-walled CNTs (MWNTs) act synergistically together when exposed to NIR to thermally ablate cancer cells.

**Conclusion:**

Given that POSS-PCU has already been used in human in first-in-man studies as trachea, lacrimal duct, bypass graft and other organs, our long-term goal is to take POSS-PCU coated CNTs to clinical studies to address the treatment of cancer by optimizing its therapeutic index and increasing its specificity via antibody conjugation.

## Background

**Figure 1:**
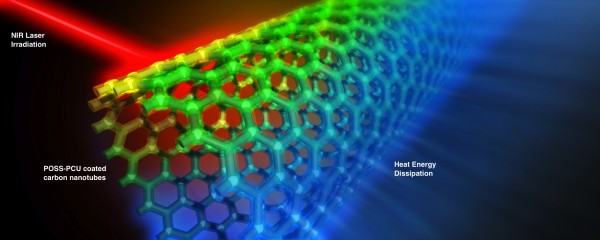
**A computer graphics rendering of photothermal ablation when POSS-PCU-functionalized CNTs are exposed to near infrared (NIR) laser.** CNTs are functionalized with a novel nanocomposite polymer, POSS-PCU, depicted as a translucent “coating” on the CNT wall. Intense heat energy is dissipated, depicted by blue emission, which can be exploited to thermally ablate cancer cells.

Carbon nanotubes (CNTs) were initially described as “helical microtubules of graphitic carbon” by Sumio Ijima (of NEC Corporation), in a letter to Nature in 1991 [1]. An unprecedented interest in its application has arisen since then, with wide-ranging potentials from being high-power energy storage devices [2], to therapeutic and diagnostic agents in cancer biology [3,4]. CNTs have a high optical absorbance in the near infrared (NIR) region [5] (700 nm to 1100 nm) and subsequently dissipate a high amount of heat energy. This phenomenon can thus be exploited in a biomedical setting to destroy cancer cells via photothermal ablation [6,7] (Figure 1).

Generally, CNTs can be classified into two broad categories: multi-walled carbon nanotubes (MWNTs) and single-walled carbon nanotubes (SWNTs). SWNTs can be conceptualized as a rolled-up sheet of planar sp^2^ hybridized carbon atoms, while MWNTs are concentric layers of SWNTs [8]. CNTs have exceptional tensile strength [9] (in the gigapascal (GPa) region), and a very high Young’s modulus [10] (in the tetrapascal (TPa) region), making them one of the strongest materials known. It has been previously reported that MWNTs display a higher thermal conductivity, compared to SWNTs, as their larger diameters allow a greater number of phonon modes, resulting in longer phonon mean free paths [11]. Due to the lack of endogenous chromophores that absorb within the NIR spectrum, biological membranes are highly transparent to NIR within wavelengths of 700 nm to 1100 nm [12]. Alluding to their electronic band structures, CNTs can attain a very high temperature (of up to 70°C) within minutes of exposure to NIR lasers [13]. Exposure of CNTs to NIR laser would release a significant amount of vibrational energy, which consequently causes cell death via coagulative necrosis. This event is known as photothermal cancer ablation, with rupturing cell membranes and denaturation of cellular proteins.

We have previously developed a novel nanocomposite polymer called polyhedral oligomeric silsesquioxane poly (carbonate-urea) urethane (POSS-PCU, trade name UCL Nano™). POSS-PCU is biocompatible [14], resistant to degradation [15], and has potential applications for being scaffolds for artificial organs [16], and coatings for medical devices [17]. Indeed, first-in-man clinical studies have already been conducted using POSS-PCU constructed three-dimensional (3D) biomimetic scaffolds of trachea [18], lacrimal duct and bypass graft, with favorable results.

A major concern for the use of CNTs in biological systems is its insolubility and inherent toxicity. With proper functionalization, for example with polyethylene glycol (PEG), CNTs appear to be well-tolerated in animal studies [19]. Hence, we postulate the amphiphilic nature of POSS-PCU would increase dispersion of CNTs in biological systems [20], as well as conferring biocompatibility. Furthermore, we also set out to investigate if the increased dispersion of CNTs would affect temperature changes during NIR laser exposure, as well as the effects of photothermal ablation on a colorectal cancer cell line.

## Results & discussion

### Conferring solubility and biocompatibility via POSS-PCU functionalization

**Figure 2:**
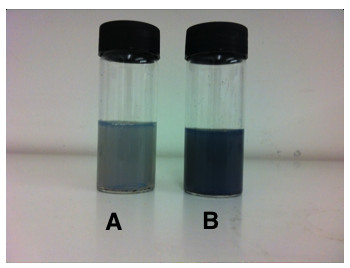
**Visual appearance of CNTs after functionalization with POSS-PCU. A** contains CNTs functionalized with 50% POSS-PCU, while **B** contains 100% POSS-PCU. Non-covalent functionalization with POSS-PCU via bath sonication confers solubility to CNTs.

**Figure 3:**
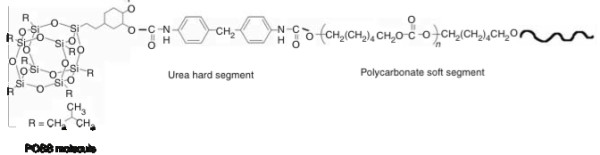
**Skeletal formula of POSS-PCU.** This novel nanocomposite polymer displays superior biocompatibility and has been used in constructing scaffolds for artificial organs as well as protective coating for medical devices.

**Figure 4:**
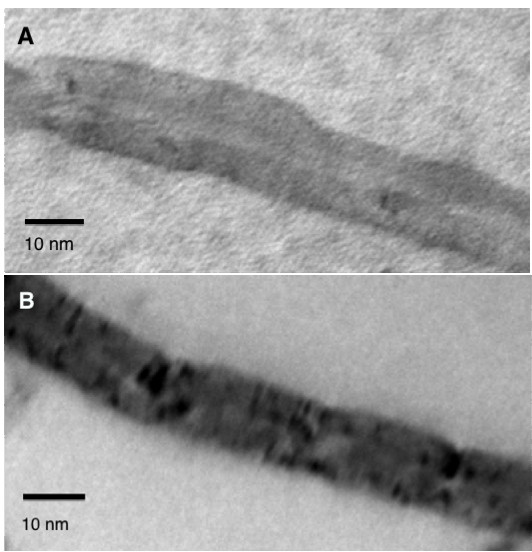
**Transmission electron microscopy of CNTs. A** shows pristine CNTs (before functionalization), **B** shows POSS-PCU coated CNTs (after functionalization).

**Figure 5:**
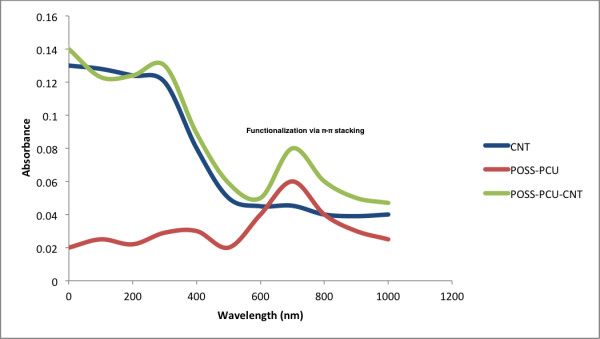
**Absorption spectra of POSS-PCU-CNT.** At a wavelength of 700 nm, POSS-PCU shows greatest absorbance (red). Non-covalent functionalization of POSS-PCU onto CNTs results in a higher absorbance at 700 nm (green), compared to unfunctionalized CNTs (blue).

Raw CNTs appear as a black powder-like substance, which is insoluble in water. Upon functionalization with POSS-PCU via bath sonication, the final product appears as a homogenous mixture (Figure 2) suitable for biological applications. The 2 main functionalization schemes are covalent and non-covalent. Covalent functionalization involves oxidation or cycloaddition reactions, which introduces functional groups onto the side-walls of CNTs [13]. However, the intrinsic chemical harshness of covalent functionalization disrupts the structure of CNTs, interrupting its inherent attributes like Raman scattering and photoluminescence [21]. In contrast, non-covalent functionalization preserves the structural integrity of CNTs, as it simply provides an external “coating”. Non-covalent functionalization occurs via hydrophobic interactions or π-π stacking of the aromatic rings [22]. Functionalization via PEG phospholipids (PL-PEG) can render CNTs biocompatible while simultaneously increasing its circulation time in biological systems [23]. Arising from this, we postulate that POSS-PCU would similarly interface with CNTs via π-π stacking of its aromatic rings of the urea hard segment (Figure 3) onto the wall of CNTs. TEM images and UV/Vis/NIR analysis (Figures 4 and 5) confirms the non-covalent functionalization of POSS-PCU onto the surface of CNTs.

Raw CNTs are insoluble in water and biological systems. Upon non-covalent functionalization with POSS-PCU, solubility was conferred, rendering it ready for use in biological systems. POSS-PCU is highly compatible, and its use in humans as a bioartificial trachea, bypass graft and lacrimal duct underscores its potential usability as a coating agent for nanoparticles as well. Unfunctionalized CNTs are not appropriate for use in biological systems, and we postulate that biocompatibility can be conferred via functionalization with POSS-PCU.

### Synergistic thermal effects of POSS-PCU and CNTs

As POSS-PCU has thermoplastic behavior, does not absorb in the near infrared spectrum. Thus, when exposed to NIR laser, the temperature of POSS-PCU remains constant at room temperature. Our results reveal that the presence of POSS-PCU does not simply maintain the temperature profile curve (as one might expect due to its absence of heating effect), but instead increases the temperature profile curve when functionalized with CNTs.

**Figure 6:**
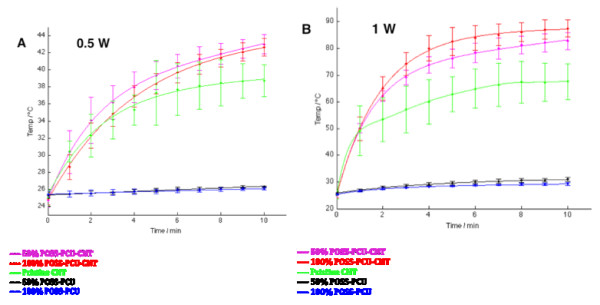
**Synergistic thermal effects of POSS-PCU and CNTs.** At both wattages (0.1 W and 1 W), POSS-PCU-CNT displays a higher rate of temperature increase and final temperature. POSS-PCU does not absorb in the NIR spectrum, but synergistically elevates the temperature profile of CNTs upon functionalization. At higher wattage, functionalization with 100% POSS-PCU shows a greater increase in temperature compared to 50% POSS-PCU.

Upon functionalization with POSS-PCU, CNTs were able to attain a greater rate of temperature increase as well as a higher final temperature of 88°C within ten minutes of NIR laser exposure compared to non-functionalized CNTs (p = 0.0006). At both wattages, synergistic thermal effects were observed, as POSS-PCU-CNT was able to attain a higher temperature than POSS-PCU and CNTs alone (Figure 6). At higher wattage, functionalization with 100% POSS-PCU shows a greater increase in temperature compared to 50% POSS-PCU. This is possibly due to the significant greater magnitude of vibrational energy, which manifests itself more synergistically at 100% POSS-PCU functionalization due to its dispersion effects. 100% POSS-PCU functionalization ensures that a greater surface area of CNTs are exposed to NIR, hence increasing its heating effects.

**Figure 7:**
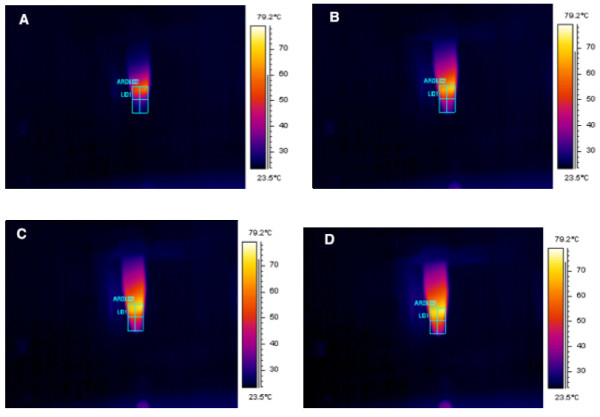
**Thermograph of NIR irradiated POSS-PCU-CNT.** POSS-PCU-CNT have a higher heating profile at 2.5 min (**A**), 5 min (**B**), 7.5 min (**C**), and 10 min (**D**), compared to unfunctionalized CNTs.

We have therefore successfully demonstrated that POSS-PCU can not only confer solubility to CNTs, but also enhance its heating effects (Figure 7). This is attributed to POSS-PCU acting like a surfactant when functionalized to CNTs, increasing its dispersion in biological systems [20]. Consequently, the surface area to volume ratio of functionalized CNTs would be increased, allowing more particles per unit area to absorb the NIR laser, thereby producing a higher rate of temperature increase as well as a higher final temperature.

### Enhanced cell kill using POSS-PCU-CNT complex

**Figure 8:**
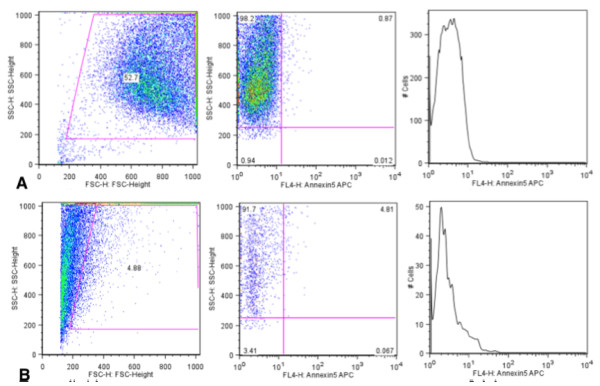
**Increased cell kill after functionalization with POSS-PCU.** Flow cytometry results reveal that 47.3% (**A**) of cells were destroyed when NIR is applied to pristine CNTs. Upon functionalization with POSS-PCU, a synergistic effect is observed, increasing cell kill to 95.12% (**B**).

**Figure 9:**
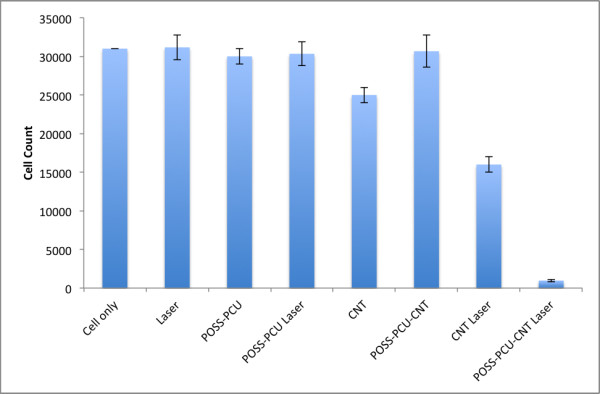
**Effects of various treatment reagents on cancer cells.** Pristine CNTs display a certain degree of toxicity to cells. Upon functionalization with POSS-PCU, CNTs do not appear to be toxic to cancer cells. POSS-PCU-CNTs are able to elicit a highly significant degree of cell kill compared to non-functionalized CNTs (p = 0.0006).

With the observation of increased thermal response to NIR exposure, we proceeded to investigate if POSS-PCU-CNT was able to thermally ablate cancer cells. A HT-29 human colorectal cancer cell line was used as a model for photothermal ablation. There was a slight decrease in cell number after incubation with CNTs (without laser), possibly due to the inherent toxicity of CNTs. In contrast, treatment with POSS-PCU-CNT (without laser) did not result in any significant change in cell number, indicating that functionalization with POSS-PCU had conferred a high degree of biocompatibility to CNTs. Concurrent with established literature, a decrease in cell number (of around 50%) was observed when (unfunctionalized) CNTs were irradiated with NIR laser. More importantly, when POSS-PCU-CNT was irradiated with NIR laser, there was a dramatic decrease in cell numbers, thermally ablating 95% of cancer cells (p = 0.0000842) (Figures 8 and 9). Hence, we have shown that POSS-PCU exerts a dual function: firstly by providing biocompatibility to CNTs, and secondly by acting synergistically with CNTs during exposure to NIR laser, resulting in a greater temperature increase and enhanced cell death.

We have successfully demonstrated that POSS-PCU-functionalized CNTs have the ability to thermally ablate cancer cells when exposed to NIR laser. Furthermore, POSS-PCU-functionalized CNTs attain a higher maximum temperature as well as a higher cell death rate compared to unfunctionalized CNTs. This has significant implications in photothermal ablative techniques, as it shows that cell death would only occur when functionalized CNTs are exposed to NIR. It has to be noted that the depth of penetration of NIR is only effect up to a few centimeters; hence for non-superficial cancers, delivery of NIR can be achieved using optical fibers. Although the toxicology of CNTs due to its size is also a current point of contention, current scientific consensus dictates that functionalizing CNTs with biocompatible molecules can confer biocompatibility to CNTS. We have demonstrated a proof-of-concept that POSS-PCU nanocomposite polymers can render CNTs biocompatible as well as augmenting its intended clinical function of photothermal cancer ablation.

## Conclusions

In this study, we present a model of synergism between CNTs and a novel nanocomposite polymer, POSS-PCU. This manifests itself in terms of temperature increase and the ability to thermally ablate cancer cells when exposed to NIR laser. Furthermore, POSS-PCU confers solubility and biocompatibility to CNTs, making it an ideal functionalizing agent to address cancer. Future work regarding cancer-specific antibody conjugation for targeted therapy and *in vivo* studies would not only be imperative, but integral for elucidating the biological applications for CNTs and nanocomposite polymers. Taken together, this work underscores the dual functionality and synergistic effects of POSS-PCU functionalization, which could have implications and ramifications in a wide-ranging area of nanomedicine.

## Materials & methods

### Carbon nanotubes (CNTs) and dunctionalization with POSS-PCU

Pristine multi-walled carbon nanotubes (MWNTs) were obtained from Nanothinx S.A. (Rio-Patras, Greece). They had a mean diameter of 15 nm, and a mean length of 100 nm. The preparation of POSS-PCU has been described elsewhere[24]. Briefly, 1 mg of MWNT was mixed with 1 mg of POSS-PCU (50% and 100%) in a glass scintillation vial and 5 ml of water was added. The resultant mixture was sonicated in a sonicator bath (Grant Instruments, Cambridge, UK) for 60 minutes at room temperature.

### Characterization of POSS-PCU-CNT Complexes

Visual characterization was conducted using a JEM-2100 F transmission electron microscope (JEOL Ltd. UK). Optical absorbance of CNT, POSS-PCU, and POSS-PCU-CNT was obtained using a LAMBDA 1050 UV/Vis/NIR (PerkinElmer, UK). Scan parameters were as follows: start (4000), number of scans (100), resolution (4), units (%T), end (400), interval (4), shuttle (interleaved). The absorption spectrum was obtained via a second derivative and a 16-point reading.

### Near infrared radiation (NIR) laser system and temperature tracking

A NIR laser system (Thorlabs Ltd, UK) with a 808 nm laser diode was used to irradiate CNTs. The laser diode controller was set to either 2300 mA (1 W) or 1250 mA (0.5 W). To prevent over-heating, the temperature controller was set to 10 kΩ. Temperature tracking and thermal images were obtained using a Thermacam™ SC500 thermal camera (FLIR Systems, UK). CNTs were exposed to NIR irradiation for 10 minutes, and temperature changes were recorded every minute.

### Thermal ablation of cancer cells

A HT-29 human colorectal cancer cell line was used as a model for photothermal cancer ablation. Briefly, 3 million (3 × 10^6^) cells were counted and seeded on a 96-well plate, with a theoretical cell count of 300,000 in each well. To each well, 100 μl of treatment reagent was added (CNT, POSS-PCU, or POSS-PCU-CNT). All treatments were done in triplicates. NIR laser was then applied for 10 minutes. Cells were then incubated at 37°C with 5% CO_2_ for 24 hours, and counted thereafter. All reagents pertaining to cell work were purchased from Invitrogen. Cell count was done using a MACSQuant Analyzer flow cytometry (Miltenyi Biotec Ltd. Surrey, UK).

### Curve fitting and statistical analyses

Curve fitting (least squares method) and statistical analyses were conducted at a 95% confidence interval using MATLAB® (MathWorks Inc, USA). Statistical significance testing was conducted using unpaired Student’s *t*-test. P values of < 0.05 were considered statistically significant.

## Competing interests

The authors declare that they have not competing interests.

## Authors’ contributions

AT, SYM, JR, GP planned, carried out, and wrote the manuscript. AMS conceived the study, participated in the design and coordination, and helped draft the manuscript. All authors read and approved the final manuscript.
